# Kondo interaction in FeTe and its potential role in the magnetic order

**DOI:** 10.1038/s41467-023-39827-1

**Published:** 2023-07-12

**Authors:** Younsik Kim, Min-Seok Kim, Dongwook Kim, Minjae Kim, Minsoo Kim, Cheng-Maw Cheng, Joonyoung Choi, Saegyeol Jung, Donghui Lu, Jong Hyuk Kim, Soohyun Cho, Dongjoon Song, Dongjin Oh, Li Yu, Young Jai Choi, Hyeong-Do Kim, Jung Hoon Han, Younjung Jo, Ji Hoon Shim, Jungpil Seo, Soonsang Huh, Changyoung Kim

**Affiliations:** 1 Center for Correlated Electron Systems, Institute for Basic Science, Seoul, 08826 Korea; 2 Department of Physics & Astronomy, Seoul National University, Seoul, 08826 Korea; 3 Department of Emerging Materials Science, DGIST, Daegu, 42988 Korea; 4 Department of Chemistry, Pohang University of Science and Technology (POSTECH), Pohang, 37673 Korea; 5 Korea Institute for Advanced Study, Seoul, 02455 Korea; 6 National Synchrotron Radiation Research Center, Hsinchu, 30076 Taiwan; 7 Department of Physics, Kyungpook National University, Daegu, 41566 Korea; 8 Stanford Synchrotron Radiation Light Source, SLAC National Accelerator Laboratory, Menlo Park, CA 94025 USA; 9 Department of Physics, Yonsei University, Seoul, 03021 Korea; 10 Center for Excellence in Superconducting Electronics, State Key Laboratory of Functional Materials for Informatics, Shanghai Institute of Microsystem and Information Technology, Chinese Academy of Sciences, 200050 Shanghai, China; 11 Beijing National Laboratory for Condensed Matter Physics and Institute of Physics, Chinese Academy of Sciences, 100190 Beijing, China; 12 School of Physical Sciences, University of Chinese Academy of Sciences, 100049 Beijing, China; 13 Songshan Lake Materials Laboratory, 523808 Dongguan, Guangdong China; 14 XFEL Beamline Division, Pohang Accelerator Laboratory, Pohang, 37673 Korea; 15 Department of Physics, Sungkyunkwan University, Suwon, 16419 Korea; 16 Present Address: Department of Physics, Massachusetts Institute of Technology, Cambridge, MA 02139 USA

**Keywords:** Electronic properties and materials, Magnetic properties and materials

## Abstract

Finding *d*-electron heavy fermion states has been an important topic as the diversity in *d*-electron materials can lead to many exotic Kondo effect-related phenomena or new states of matter such as correlation-driven topological Kondo insulator. Yet, obtaining direct spectroscopic evidence for a d-electron heavy fermion system has been elusive to date. Here, we report the observation of Kondo lattice behavior in an antiferromagnetic metal, FeTe, via angle-resolved photoemission spectroscopy, scanning tunneling spectroscopy and transport property measurements. The Kondo lattice behavior is represented by the emergence of a sharp quasiparticle and Fano-type tunneling spectra at low temperatures. The transport property measurements confirm the low-temperature Fermi liquid behavior and reveal successive coherent-incoherent crossover upon increasing temperature. We interpret the Kondo lattice behavior as a result of hybridization between localized Fe 3d_xy_ and itinerant Te 5p_z_ orbitals. Our observations strongly suggest unusual cooperation between Kondo lattice behavior and long-range magnetic order.

## Abstract (web summary)

The Kondo hybridization typically occurs in heavy-fermion systems containing f electrons, although recently it has been reported in d-electron systems. Kim et al. report spectroscopic evidence of the Kondo hybridization in FeTe and discuss it role in the mechanism of the magnetic order.

## Introduction

Coupling between spin and electronic degrees of freedom in condensed matter systems leads to a variety of emergent phenomena such as colossal magnetoresistance, Rashba effect, anomalous Hall effect, and unconventional superconductivity^1–5^. In particular, understanding how the spin and electronic degrees of freedom interact in such systems is the key to elucidating the underlying physical mechanism and can thus be a steppingstone to future practical applications.

One of the canonical fields to study the interplay of these degrees of freedom is heavy-fermion (HF) materials^6,7^. HF states appear as a result of the interaction between itinerant electrons and localized magnetic moments, known as Kondo interaction. Previous experimental/theoretical studies show most of the HF materials are f-electron systems^3,6,7^. It was only recently proposed that d-electron systems can also host HF states via Kondo interactions^8–11^. HF states in d-electron materials are especially important due to the possibility that the diversity of d-electron systems may result in exotic Kondo interaction-related phenomena, such as topological Kondo insulating state^12^ or cooperation between Kondo lattice behavior and long-range magnetism^13^. Thus, the novelty calls for new studies to find HF in d-electron material groups.

FeTe can be a candidate material to observe d-electron HF states. Its electron correlation is the strongest among the iron-based superconductors (IBSCs)^14^. The magnetic ground state is known to be bicollinear antiferromagnetism (BAFM) with a large magnetic moment of 2.1 μ_B_, implying its local nature of the magnetism^14^. The Sommerfeld coefficient of FeTe is reported to be 31.4 mJ/(K^2^ mol), indicating a heavy effective mass of the system^15^. This value is much larger than that of other iron chalcogenides; FeS and FeSe for instance have 3.8 and 6.9 mJ/(K^2^ mol), respectively^16,17^.

In addition to these HF-related properties, other transport properties suggest the existence of strong spin-electron interaction. The temperature-dependent resistivity exhibits a drastic change at the Néel temperature (T_N_). It shows an insulating behavior above T_N_, but a metallic behavior below T_N_^18^. The aforementioned properties of FeTe imply that the local magnetic moment significantly affects the electronic structure. Thus, electronic structure studies on the HF state of FeTe can unveil its origin and how it couples with magnetism.

Here, we report on a comprehensive study on FeTe using angle-resolved photoemission spectroscopy (ARPES), transport property measurements, and scanning tunneling spectroscopy (STS). We observe a hallmark of an HF behavior in ARPES spectra: a sharp quasiparticle peak (QP) near the Γ point and its strong temperature dependence. The observed QP is attributed to Kondo hybridization between Fe 3d_xy_ and Te 5p_z_. The Kondo hybridization scenario is further supported by STS results, showing the Fano line shape and narrow hybridization gap. In this picture, the recovery of metallic behavior in the low-temperature region is due to the emergence of the strong QP around the Γ point. We also conducted a Heisenberg model calculation, suggesting the Kondo interaction may be responsible for the emergence of BAFM in FeTe. These results provide a unified perspective that the Kondo interaction determines the exotic physical and magnetic properties in FeTe.

## Results

### Transport properties

**Fig. 1:**
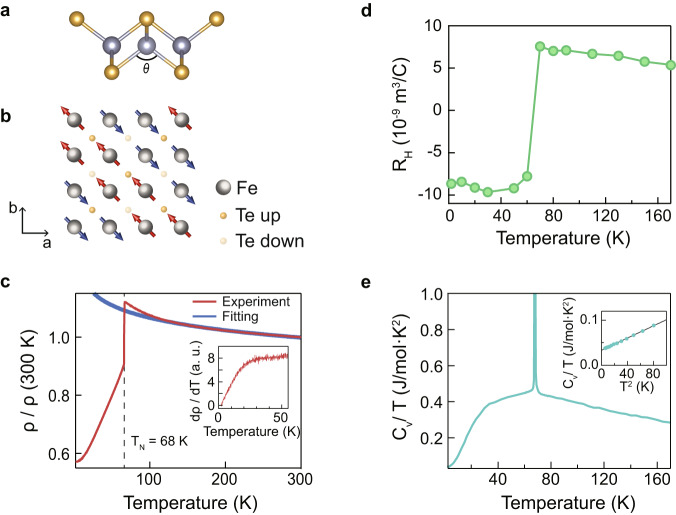
Crystal structure and transport results of FeTe. **a** Crystal structure of FeTe. **b** Spin configuration of bicollinear antiferromagnetic (BAFM) state in FeTe. **c** Temperature-dependent resistivity. The red curve is the experimental data, while the blue curve is the fitting result of the logarithmic function ($a+b{{{{{\rm{log }}}}}}(T)$) of the data between 120 and 300 K. Inset shows the temperature-derivative of the resistivity. **d** Temperature-dependent Hall co**e**fficient. **e** Temperature-dependent C_v_/T. Inset shows C_v_/T vs T^2^ plot in the low-temperature region. The black solid line in the inset is the fit result of C_v_/T = γ + βT^2^. Source data are provided as a Source Data file.

FeTe has the simplest crystal structure among the IBSCs as shown in Fig. 1a. Compared to other similar iron chalcogenide systems of FeSe and FeS, FeTe has a distinctive bonding angle value *θ* shown in Fig. 1a. More specifically, Te atom is pushed away from the Fe plane due to its large atomic size and, as a result, FeTe has a small *θ* value^19^. This aspect of the crystal structure leads to localization of the Fe 3d_xy_ band as the d_xy_ orbital is confined in the Fe plane^13,19^. A recent ARPES study showed a complete loss of coherent spectral weight in the d_xy_ band in FeTe, indicating a strong localization in the band^14,20^. The magnetic ground state of FeTe is bicollinear antiferromagnetism (BAFM) as shown in Fig. 1b below a Néel temperature of near 70 K^18^. It is noteworthy that among IBSCs, only FeTe exhibits BAFM. The ordering vector of BAFM in FeTe is (π/2, π/2) (1-Fe unit cell) while that of conventional AFM shown on other IBSCs is (π, 0)^21^.

Transport properties show a close relationship with magnetic properties. The temperature-dependent resistivity in Fig. 1c shows insulating behavior above T_N_. We find the temperature dependence follows a logarithmic behavior of -ln(T). On the other hand, it abruptly recovers a metallic behavior below T_N_. More specifically, it shows a Fermi liquid behavior below 15 K with a T^2^ dependence resistivity, and a T-linear behavior between 30 K and 70 K. These T-dependent behaviors indicate the existence of coherent-incoherent crossover around 15 K (see the inset of Fig. 1c and Supplementary Note 1). It is also noteworthy that the resistivity shows a minimum at around 2.2 K (see Supplementary Note 2 for the corresponding data and discussion). The Hall coefficient, as well as the resistivity, shows a drastic change at T_N_. The Hall coefficient changes hole dominant (T > T_N_) to electron dominant (T < T_N_) at T_N_ as can be seen in Fig. 1d. The crossover behavior seen in the resistivity data can be also found in the heat capacity data in Fig. 1e; C_v_/T deviates from T^2^ behavior around 15 K (see Supplementary Note 1 for the determination of the deviation temperature). Further analysis shows that the Sommerfeld coefficient extracted from the heat capacity is 33.4 mJ/mol K^2^ (see the inset of Fig. 1e). It is much larger than that of other iron chalcogenides. For instance, it is 3.8 and 6.9 mJ/mol K^2^ for FeS and FeSe, respectively^16,17^.

### Electronic structures

**Fig. 2:**
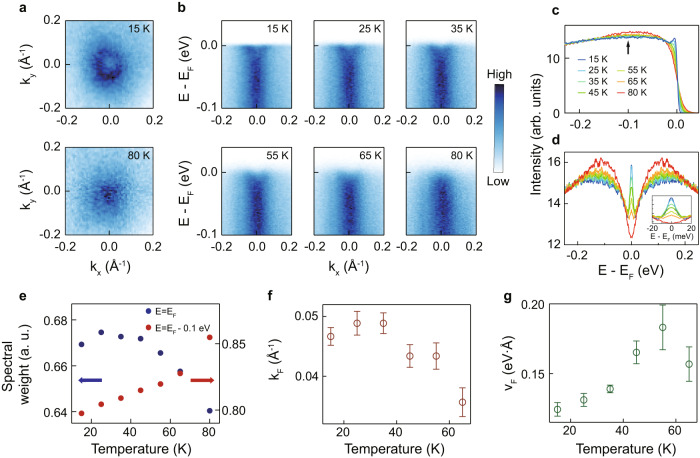
Electronic structure of FeTe. **a** Fermi surface (FS) maps from high-resolution laser ARPES measurements, obtained at 15 and 80 K. **b** Temperature-dependent high symmetry cuts along the Γ’-X’ direction. ARPES data were taken with 11 eV photons. **c** Energy distribution curves (EDCs) integrated within a certain momentum range (k_x_^2^ + k_y_^2^ < (0.15 Å^−1^)^2^). The EDCs are normalized with the integrated intensity from an energy window of −0.25 eV <E—E_F_ < −0.2 eV. **d** Symmetrized EDCs of (**c**). Inset: enlarged view of EDCs near the Fermi level. **e** Temperature-dependent spectral weight at E = E_F_ and E = E_F_ – 0.1 eV. **f**, **g** Temperature-dependent Fermi momentum (k_F_) and Fermi velocity (v_F_), respectively, obtained from momentum distribution curve (MDC) analysis. Errors bars in (**f**, **g**) represent the fitting errors of Fermi momentum and Fermi velocity, respectively. Source data are provided as a Source Data file.

We turn our attention to the electronic structure of FeTe. High-resolution laser ARPES experiments were performed to track the temperature-dependent evolution of the electronic structure. The Fermi surfaces (FSs) near the Γ’ point (corresponds to k_z_ ≈ 0.5 π/*c* where *c* is the lattice constant along the *z* direction; see “Methods” for details) shown in Fig. 2a exhibit significant temperature dependence as the temperature decreases from 80 to 15 K. A single circular FS pocket is clearly observed at 15 K while it becomes a blob at 80 K. Evolution of the electronic structure can also be seen in the high symmetry cuts along the *k*_x_-direction shown in Fig. 2b. It is revealed that the FS pocket observed at 15 K in Fig. 2a comes from an electron band. As the temperature increases, the electron band tends to be broadened and vanishes abruptly at 80 K.

This observed temperature dependence of the band can be more clearly seen in the temperature-dependent energy distribution curves (EDCs) plotted in Fig. 2c. A clear QP is observed at the lowest temperature, which comes from the electron band mentioned above. Upon increasing temperature, the QP is gradually suppressed while the spectral weight of the hump centered at −0.1 eV, indicated by an arrow in Fig. 2c, gradually increases. Such spectral weight transfer behavior is more pronounced in symmetrized EDCs in Fig. 2d. Analysis of the spectral weight transfer behavior is depicted in Fig. 2e. It clearly shows that the lost QP spectral weight is transferred to the 0.1 eV hump, demonstrating that the observed temperature dependence is intrinsic. It is also noteworthy that the full width at half maximum (FWHM) of the QP obtained from a Lorentzian fitting is 7.9 meV as can be seen in the inset of Fig. 2d, implying remarkable heavy mass and long quasiparticle lifetime of the band.

Additional band-fitting analyses provide more information about the temperature-dependent evolution of the band. We extract the Fermi momentum (k_F_) and Fermi velocity (v_F_) using momentum distribution curve (MDC) analysis as depicted in Fig. 2f, g, respectively. Temperature-dependent k_F_ value shows that the FS pocket size tends to enlarge upon cooling. Meanwhile, v_F_ of the electron band decreases with the temperature. From these results, we can infer that the temperature evolution of the k_F_ and v_F_ did not result from a simple chemical potential shift. The origin of the evolution will be discussed below.

**Fig. 3:**
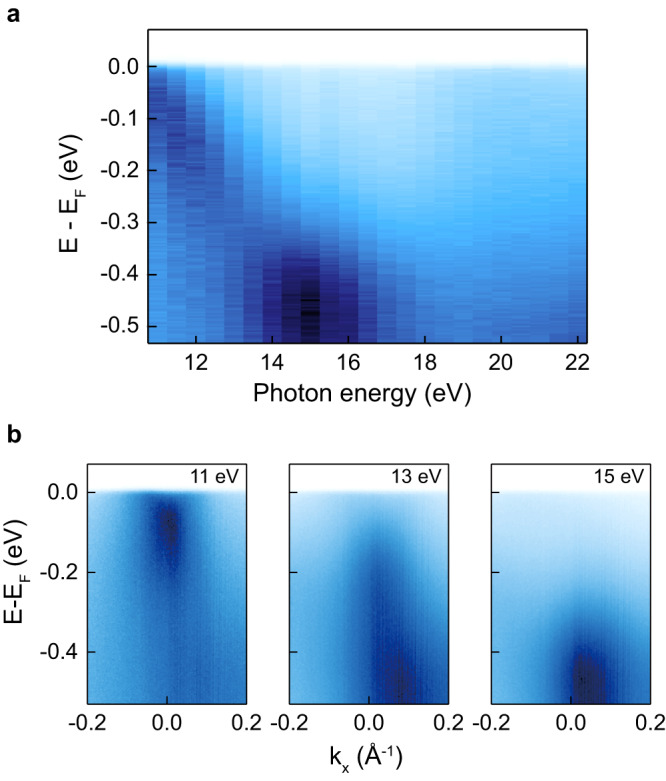
Photon energy-dependent electronic structure. **a** Photon energy-dependent electronic structure near the Γ point. **b** Photon energy-dependent high symmetry cuts along the Γ-X direction, obtained using 11, 13, 15 eV photon. Source data are provided as a Source Data file.

The photon energy-dependent ARPES result gives further insights into the origin of the band. As can be seen in Fig. 3, the electron band which is clearly visible at 11 eV has a strong k_z_ dispersion. As the photon energy increases, the band shifts to the higher binding energy side, and its energy scale becomes more than 0.5 eV. Considering FeTe is in the strongly correlated limit, a bandwidth of 0.5 eV far surpasses that of Fe 3d bands^19^. In addition, the photoionization cross section of Te 5p orbital is much larger than that of Fe 3d orbital at 11 eV^22^. Thus, the band observed at 11 eV is likely to be mostly from Te p_z_ orbital. We note that similar k_z_ dispersion behavior was also reported for FeTe_0.55_Se_0.45_^23^. Polarization- and experimental geometry-dependent ARPES measurements also confirm the p_z_ character of the band (see Supplementary Note 10).

Considering the large dispersion of the p_z_ band away from E_F_ as shown in Fig. 3, the sharp QP near E_F_ implies that the band undergoes a strong modulation. Two scenarios may be considered for the modulation: (i) electron-bosonic mode coupling and (ii) Kondo hybridization between the itinerant and localized bands. It was claimed in a previous ARPES study on FeTe that the feature is a result of strong electron-phonon coupling, namely a polaronic behavior^24^. However, such a scenario may not explain the enlargement of the Fermi surface at low temperatures in Fig. 2f since an electron-boson coupling should conserve the k_F_. Alternatively, one can consider a Kondo hybridization scenario which should also show a mass enhancement at low temperatures and strong temperature dependence of the QP. Therefore, it is highly desirable to have an alternative way to discern the two scenarios.

### Fano line shape and hybridization gap

**Fig. 4:**
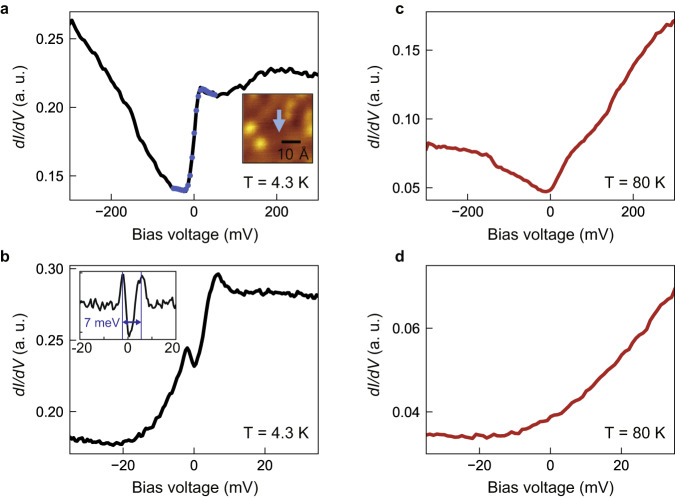
STS results on FeTe. **a** Differential conductance (d*I/*d*V*) spectrum measured on FeTe surface at 4.3 K. The blue circles represent the Fano fitting of the Kondo resonance (see Supplementary Note 4 for the fitting parameters). The inset shows the position where the spectrum is taken. *V*_bias_ = −300 mV, *I* = 100 pA, and lock-in modulation *V*_mod_ = 5 mV_pp_. **b** d*I/*d*V* spectrum enlarged around the Fermi energy. The inset is the spectrum after subtracting the smoothly varying background. *V*_bias_ = −40 mV, *I* = 100 pA and *V*_mod_ = 500 μV_pp_. **c** d*I/*d*V* spectra measured at 80 K. *V*_bias_ = −300 mV, *I* = 50 pA and *V*_mod_ = 5 mV_pp_. **d** Zoomed-in d*I/*d*V* spectrum. *V*_bias_ = −40 mV, *I* = 50 pA and *V*_mod_ = 500 μV_pp_. Source data are provided as a Source Data file.

Whether the strong renormalization of the dispersion near E_F_ is due to Kondo hybridization or not may be determined based on tunneling spectra. Shown in Fig. 4 are STS data at 4.3 and 80 K. A wide energy range scan at 4.3 K depicted in Fig. 4a shows an asymmetric spectrum. The spectrum is found to be well-fitted with a Fano line shape as illustrated in the figure. It is well-known that tunneling spectra from a Kondo singlet state should exhibit a Fano-type resonance^13,25^. The Fano fit shown as blue circles in Fig. 4a gives a Fano line width (Γ value) of 24.1 meV, which corresponds to the Kondo temperature of about 280 K. Furthermore, a closer look of the data over a narrow energy range around E_F_ plotted in Fig. 4b shows a gap feature that is consistent with a gap expected for a Kondo hybridization scenario. We subtract the smoothly varying background from the data and plot it in the inset. The subtracted data shows a gap with a size of about 7 meV as seen in Fig. 4b. In addition, it is seen that the gap feature is slightly shifted to the unoccupied side. Plotted in Fig. 4c, d are d*I/*d*V* spectra taken at 80 K, above T_N_. The two spectra are taken over the same energy ranges as the 4.3 K data. The Kondo-related features are expected to disappear at high temperatures, which are indeed seen in the high-temperature data in Fig. 4c, d; the Fano behavior is weakened and the hybridization gap has disappeared. Therefore, these observations—Fano behavior and narrow gap near E_F_—are clear signs of Kondo hybridization, confirming that FeTe exhibits Kondo hybridization below T_N_.

## Discussion

**Fig. 5:**
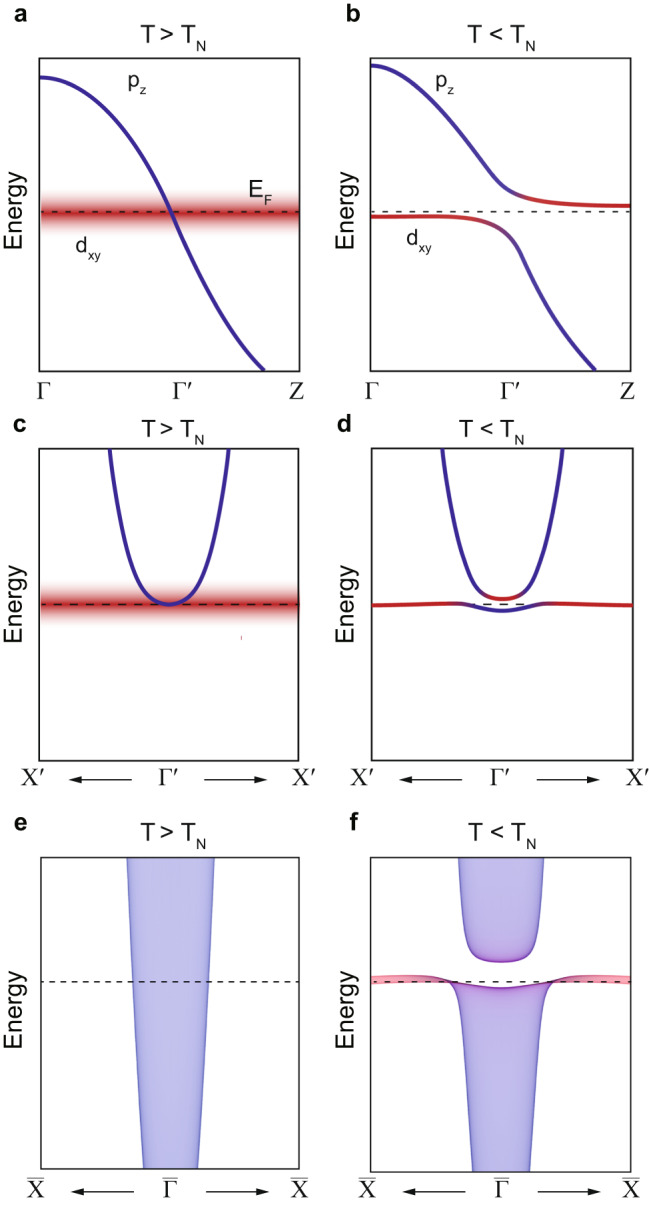
Schematic of the Kondo hybridization scenario. **a**, **b** Band structure of FeTe along the Γ-Z direction (out-of-plane) above and below T_N_, respectively. **c**, **d** Band structure of FeTe along the Γ-X direction (in-plane) above and below T_N_, respectively. **e**, **f** Simulated band structure projected onto the (001) surface along the Γ-X direction (in-plane) above and below T_N_, respectively. Blue bands denote *p*_z_ orbital, and red bands denote *d*_xy_ orbital.

Fully considering our comprehensive data, we argue that the electron band that emerges below T_N_ is a result of a Kondo hybridization between the itinerant p_z_ and localized d_xy_ bands. The argument is based on the fact that only the d_xy_ orbital of FeTe is in a localized state, which is a prerequisite for the Kondo effect^6,14,19^. Density functional theory calculations also confirm the band we measured in ARPES has Te p_z_ and Fe d_xy_ orbital characters (see Supplementary Note 8). Here, it is also noteworthy that the appearance of the coherence peak may be accounted for within the coherence-incoherence crossover picture in Hund’s metal^26,27^ as observed in some of the iron-based superconductors^28–30^. However, the Kondo hybridization picture is needed to explain the other aspects of the experimental results. Indeed, recent theoretical work proposed that the interorbital hopping in the orbital-selective Mott phase can develop a narrow quasiparticle peak near the Fermi level^31^. In this perspective, our work emphasizes the role of interorbital coupling. When the system enters the BAFM state, the p_z_ and d_xy_ bands start to Kondo hybridize as illustrated in Fig. 5; the strongly dispersive p_z_ band along k_z_ direction crosses the localized d_xy_ band, resulting in a Kondo hybridization and heavy electron band. The correlation between Kondo hybridization and BAFM is discussed later. Based on known band dispersions, we simulate the band structure with a finite hybridization between the p_z_ and d_xy_ band. The simulated band structures projected onto the (001) surface in Fig. 5e, f well coincide with ARPES results shown in Fig. 2b at the temperature of 80 K and 15 K, respectively. In addition, the narrow gap in the unoccupied side at low temperature and its disappearance at high temperature in the STS data directly support the band diagram illustrated in Fig. 5f, e, respectively. The details of the simulation are described in the Materials and Methods section. The Kondo hybridization scenario is further supported by previous inelastic neutron scattering measurements on FeTe: the study reported that the local magnetic moment of FeTe is S = 1 at 10 K but it unexpectedly grows to S = 3/2 at 300 K, suggesting low-temperature Kondo screening of the local moments by itinerant electrons^32^. Note that the d_xy_ band is not visible near the Fermi level since d_xy_ band is strongly localized, and thus its spectral weight near the Fermi level is mostly transferred to the high binding energy region, and the photoionization cross section of Te 5p orbitals far surpass that of Fe 3d orbitals at 11 eV photon^20,23^.

The observed heavy electron band resulting from Kondo hybridization can address the unique transport properties of FeTe: (i) recovery of metallic behavior below T_N_, (ii) sudden sign change in the Hall conductivity at T_N_, and (iii) emergent Fermi liquid behavior at low temperature. First, the recovery of metallic behavior can be understood through the emergence of the sharp and strong QP at the Fermi level near the Γ point at T_N_; the transport properties are dominated by the QP. The emergence of the electron QP below T_N_ can also explain the sign change in the Hall conductivity, from hole dominant (T > T_N_) to electron dominant (T < T_N_). A previous study reported that recovery of the metallic behavior and Hall coefficient change may be related to the formation of pseudogap near the Brillouin zone corner^33^. However, their observation is not enough to explain the abrupt change in the resistivity and Hall conductivity. It is also noteworthy that such a strong QP and its strong temperature dependence are only observed at the Γ point (see Supplementary Note 3 for the temperature-dependent ARPES results on the X point pocket.). Thus, we believe the FS near the Γ point, which exhibits a sudden change at T_N_, dominates transport properties. Finally, the sharp QP bandwidth of 7.9 meV indicates a long quasiparticle lifetime, indicating that FeTe is in a Fermi liquid regime at low temperatures. This observation is consistent with the unique transport results and enhanced Sommerfeld coefficient of FeTe. We note that recent ARPES and STS studies on CeRh_2_Si_2_ and SmB_6_ reported significantly different Kondo properties at the surface and in the bulk^34–36^. In such cases, considering the surface sensitivity of ARPES and STS, the Kondo-related properties of FeTe observed via ARPES and STS can be different from those of transport measurements. However, the crystal structure of FeTe is quasi-two-dimensional, which is distinct from CeRh_2_Si_2_ and SmB_6_^20^. This feature might be the reason for the consistency in the Kondo properties of FeTe observed by ARPES and transport measurements.

The overall temperature dependence of electronic structures and transport properties are well explained within the Kondo lattice scenario. In the paramagnetic (PM) state, FeTe is in the Kondo scattering regime, consistent with the logarithmic resistivity^37,38^ and estimated Kondo temperature from Fano line width (see Supplementary Note 4 for detailed parameters). From the electronic structure point of view, the strong scattering in the Kondo scattering regime results in the breakdown of a well-defined quasiparticle, which in turn leads to loss of spectral weight and its transfer to a higher binding energy region^37,39^. Thus, the hump structure is the incoherent counterpart of the QP, supported by the spectral weight transfer as shown in Fig. 2e. The broadened but persistent Fano line shape at 80 K also indicates the system is still in the Kondo scattering regime, while strongly suppressed coherency above T_N_ leads to the loss of the QP. On the other hand, when the system enters the BAFM state, low-temperature behaviors of a Kondo lattice emerge: a sharp quasiparticle peak in the electronic structure induced by Kondo hybridization^35,37^ as well as a Fermi liquid behavior (T^2^ dependence) at low temperature followed by a coherent-incoherent crossover in resistivity^8,13,37^. Based on these facts, we may address the unique feature of the Kondo lattice behavior in FeTe; low-temperature Kondo lattice behaviors in FeTe suddenly set in at the onset of BAFM as evidenced by the abrupt drop in the resistivity and sudden emergence of QP at T_N_. This drastic shift of the system to the low-temperature Kondo lattice regime at the onset of the BAFM suggests a possible positive correlation between BAFM and Kondo lattice behavior in FeTe.

**Fig. 6:**
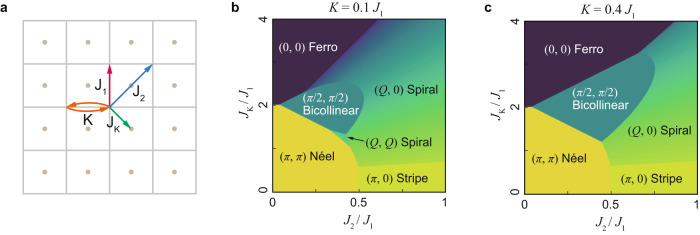
Magnetic phase diagram of FeTe from Heisenberg model. **a** Definition of the Heisenberg model parameters. Gray solid lines denote the prime square lattice, whereas brown dots denote the sublattice. J_1_ and J_2_ are nearest-neighbor (NN) and next nearest-neighbor (NNN) exchange interactions, respectively, on the prime lattice. J_K_ denotes NN exchange interaction between the prime lattice and sublattice. *K* is the NN biquadratic exchange interaction. **b**, **c** Magnetic phase diagram calculated from the model Hamiltonian (Eq. (1)) with *K* = 0.1 and 0.4, respectively.

To reveal the underlying mechanism of the positive correlation between BAFM and Kondo lattice behavior in FeTe, we conducted a Heisenberg model calculation with an additional Fe–Te exchange interaction. Based on the established two-neighbor Heisenberg model with the biquadratic term (J_1_–J_2_–K model) on a prime square lattice^40–43^, we additionally introduce a centered sublattice as shown in Fig. 5a to take into account the Fe–Te interaction (defined as J_K_ hereafter). We define the J_1_–J_2_–J_K_–K model on the combined lattice as

1 $$H={J}_{1}\mathop{\sum}\limits_{ < i,j > }\vec{{S}_{i}}\cdot \vec{{S}_{j}}-K\mathop{\sum}\limits_{ < i,j > }{\left(\vec{{S}_{i}}\cdot \vec{{S}_{j}}\right)}^{2}+{J}_{2}\mathop{\sum}\limits_{\ll i,j\gg }\vec{{S}_{i}}\cdot \vec{{S}_{j}}+{J}_{K}\mathop{\sum}\limits_{ < i,k > }\vec{{S}_{i}}\cdot \vec{{s}_{k}}$$

where J_1_ and J_2_ are nearest-neighbor (NN) and next nearest-neighbor (NNN) exchange interactions on the prime lattice, respectively, and K is the NN biquadratic exchange interaction, while J_K_ is the NN interaction between prime lattice and sublattice as described in Fig. 6a. *i* and *j* are indices for the prime lattice, and *k* is the sublattice index.

We solved the J_1_–J_2_–J_K_–K model for various K values and obtained the corresponding magnetic phase diagram in Fig. 6b, c. For a small J_K_, the model well reproduces (π, 0) stripe phase in iron pnictides. As J_K_ grows, (π/2, π/2) BAFM starts to be stabilized and spans the phase diagram over a wide range of K (see Supplementary Fig. 7 for an extended phase diagram.). Within the J_K_-induced BAFM scenario, the sublattice (Te atom for FeTe) should be also spin-polarized accordingly. We note that previous spin-polarized scanning tunneling microscopy measurements on FeTe revealed that Te atoms are also spin-polarized in the BAFM state^44^. These results suggest that J_K_, an exchange interaction between Fe and Te, may play a crucial role in stabilizing the BAFM in FeTe. This J_K_-induced BAFM scenario thus explains the positive correlation between Kondo lattice behavior and BAFM since the Kondo lattice behavior and BAFM share the same origin, J_K_. The positive correlation between long-range magnetism and Kondo lattice state is reminiscent of the underscreened Kondo lattice model in UTe and UCu_0.9_Sb_2_, where a local magnetic moment of S = 1 is not fully screened by itinerant electrons^43,44^. Likewise, the local moment of S = 3/2 in FeTe at 300 K is not fully screened, resulting in a residual local moment of S = 1 at 10 K^32^, suggesting a possible analogy with the underscreened Kondo lattice model^45,46^.

We find the J_1_–J_2_–J_K_–K model has further implications. It was previously reported that an unexpected ferromagnetic (FM) state emerges under hydrostatic pressure^47^. A transition from BAFM to FM occurs in our calculated magnetic phase diagram if J_K_ is further increased. Note that previously proposed Heisenberg models had to employ the third nearest-neighbor exchange interaction (J_3_) to account for the BAFM in FeTe, but could not predict the FM phase^42,43^. In other words, the inclusion of J_K_ may be the key to understanding the magnetic order in FeTe.

Recently, there have been numerous studies reporting that orbital selectiveness is a prominent ingredient to make physics diverse in correlated d-electron multiorbital systems^14,28,29,48–50^. In particular, while the orbital-selective Mott phase itself is an intriguing phenomenon, another important aspect is that materials with orbital-selective Mott phase are vulnerable to Kondo hybridization and thus may result in a new type of HF state^8–10^. We thus suppose that the local magnetic moment formed in the orbital-selective Mott phase critically affects the physical and magnetic properties of FeTe via Kondo interaction^14^. Our results shed light on the role of the local magnetic moments in correlated d-electron multiorbital systems.

## Methods

### Sample growth and characterization

Single crystals of FeTe were synthesized using a modified Bridgman method^51^. Stoichiometric iron (99.99%) and tellurium (99.999%) were sealed into an evacuated quartz tube and placed in a two-zone furnace. The hot (cold)-zone of the furnace was set to be 1070 (970) °C and slowly cooled down to 570 (470) °C at a rate of 2 °C/h. The estimated excess iron concentration of Fe_1+y_Te is in the range between 0.08 and 0.12, which is determined from STM topography and inductively coupled plasma measurements.

### ARPES measurements

High-resolution ARPES measurements were performed with a home lab-based laser ARPES system equipped with a 10.897 eV laser (UV-2 from Lumeras) and a time-of-flight analyzer (ARTOF 10k from Scienta Omicron)^52^. The k_z_ of the Γ’ point is approximately determined to be 0.5 π/*c*, with the photon energy of 11 eV and inner potential of 13 eV^53^. Photon energy-dependent ARPES measurements were performed at BL-21B1 of the National Synchrotron Radiation Research Center (NSRRC). All ARPES measurements were conducted with p-polarized light. Overall energy resolution for the laser ARPES and photon energy-dependent ARPES measurements was set to be 2 and 14 meV, respectively. The temperature-dependent measurements were conducted upon cooling, starting from 80 K. The photon energy-dependent measurements were conducted at 15 K.

### Transport measurements

The resistivity and heat capacity measurements were carried out with a Physical Property Measurement System (PPMS from Quantum Design). The resistivity and Hall coefficient measurement was conducted in a standard 4-probe and Hall bar geometry, respectively.

### STM measurements

STM experiments have been performed using a home-built low-temperature STM operating at 4.3 K or 80 K. The FeTe single crystal precooled to 15 K was cleaved in the ultrahigh vacuum condition. The cleaved FeTe sample was immediately inserted into the STM head. A PtIr tip is used for the measurements, and the tip quality is checked by the surface interference pattern on Cu(111). To acquire d*I/*d*V* spectra, a standard lock-in technique was used with a modulation frequency of *f* = 718 Hz.

### Band structure simulation

The band structure simulation with a toy model is conducted to simulate ARPES results with finite k_z_ broadening where a strongly k_z_-dispersive band is hybridized with a localized band. The simulation is based on a two-band model with a finite hybridization. The Hamiltonian is defined as

$$H=\left(\begin{array}{cc}{E}_{p}(\vec{k}) & \varDelta \\ \varDelta & {E}_{d}(\vec{k})\end{array}\right),$$

where

$${E}_{p}\left(\vec{k}\right)=5t{\left(\frac{{k}_{x}}{\pi }\right)}^{2}+100t{{{{{\rm{cos }}}}}}\left({k}_{z}\right)-\,\mu,$$

$${E}_{d}\left(\vec{k}\right)=-\frac{t}{200}{\left(\frac{{k}_{x}}{\pi }\right)}^{2}-t{{{{{\rm{cos }}}}}}\left({k}_{z}\right)\,-\,\mu,$$

$$\varDelta=10t.$$

*t* is the energy scale of the hopping parameter, and μ is the chemical potential of the system which is set arbitrarily. The basis of each axis is p, d orbitals, respectively. The in-plane dispersion is defined as parabolic and out-of-plane dispersion is defined as a cosine function. The dispersion parameter is based on the DFT calculation and ARPES results on FeTe_1-x_Se_x_^20,22–24^. The diagonalized band structures are projected onto the (001) surface and plotted in Fig. 5f. For Fig. 5e, only ${E}_{p}\left(\vec{k}\right)$ is plotted to simulate the ARPES data at 80 K where hybridization does not occur. The blue and red intensity in Fig. 5 denotes the orbital character of p_z_ and d_xy_, respectively.

### Supplementary information

Supplementary Information

Peer Review File

## Data Availability

Source data are available at 10.6084/m9.figshare.23538054. Other data that support the findings of this study are available from the corresponding author upon reasonable request.
