# Author Correction: Kondo interaction in FeTe and its potential role in the magnetic order

**DOI:** 10.1038/s41467-024-46779-7

**Published:** 2024-03-18

**Authors:** Younsik Kim, Min-Seok Kim, Dongwook Kim, Minjae Kim, Minsoo Kim, Cheng-Maw Cheng, Joonyoung Choi, Saegyeol Jung, Donghui Lu, Jong Hyuk Kim, Soohyun Cho, Dongjoon Song, Dongjin Oh, Li Yu, Young Jai Choi, Hyeong-Do Kim, Jung Hoon Han, Younjung Jo, Ji Hoon Shim, Jungpil Seo, Soonsang Huh, Changyoung Kim

**Affiliations:** 1 Center for Correlated Electron Systems, Institute for Basic Science, Seoul, 08826 Korea; 2 Department of Physics & Astronomy, Seoul National University, Seoul, 08826 Korea; 3 Department of Emerging Materials Science, DGIST, Daegu, 42988 Korea; 4 Department of Chemistry, Pohang University of Science and Technology (POSTECH), Pohang, 37673 Korea; 5 Korea Institute for Advanced Study, Seoul, 02455 Korea; 6 National Synchrotron Radiation Research Center, Hsinchu, 30076 Taiwan; 7 Department of Physics, Kyungpook National University, Daegu, 41566 Korea; 8 Stanford Synchrotron Radiation Light Source, SLAC National Accelerator Laboratory, Menlo Park, CA 94025 USA; 9 Department of Physics, Yonsei University, Seoul, 03021 Korea; 10 Center for Excellence in Superconducting Electronics, State Key Laboratory of Functional Materials for Informatics, Shanghai Institute of Microsystem and Information Technology, Chinese Academy of Sciences, 200050 Shanghai, China; 11 Beijing National Laboratory for Condensed Matter Physics and Institute of Physics, Chinese Academy of Sciences, 100190 Beijing, China; 12 School of Physical Sciences, University of Chinese Academy of Sciences, 100049 Beijing, China; 13 Songshan Lake Materials Laboratory, 523808 Dongguan, Guangdong China; 14 XFEL Beamline Division, Pohang Accelerator Laboratory, Pohang, 37673 Korea; 15 Department of Physics, Sungkyunkwan University, Suwon, 16419 Korea; 16 Present Address: Department of Physics, Massachusetts Institute of Technology, Cambridge, MA 02139 USA

**Keywords:** Electronic properties and materials, Magnetic properties and materials

Correction to: *Nature Communications* 10.1038/s41467-023-39827-1, published online 12 July 2023

In this article the affiliation details for Young Jai Choi were incorrectly given as ‘Stanford Synchrotron Radiation Light Source, SLAC National Accelerator Laboratory, Menlo Park, CA 94025, USA’ but should have been ‘Department of Physics, Yonsei University, Seoul 03021, Korea’. The original article has been corrected.

