# Supplementary material for: Kondo interaction in FeTe and its potential role in the magnetic order
Source: Nat Commun. 2023 Jul 12;14:4145. doi: 10.1038/s41467-023-39827-1 (PMC10338437; doi:10.1038/s41467-023-39827-1)
Supplement: Supplementary file 1 — Supplementary Information [file 41467_2023_39827_MOESM1_ESM.pdf]

Supplementary Information for

Kondo interaction in FeTe and its potential role in the magnetic order

Younsik Kim *et al.*

Supplementary Note 1: Coherence-incoherence crossover seen in transport measurements

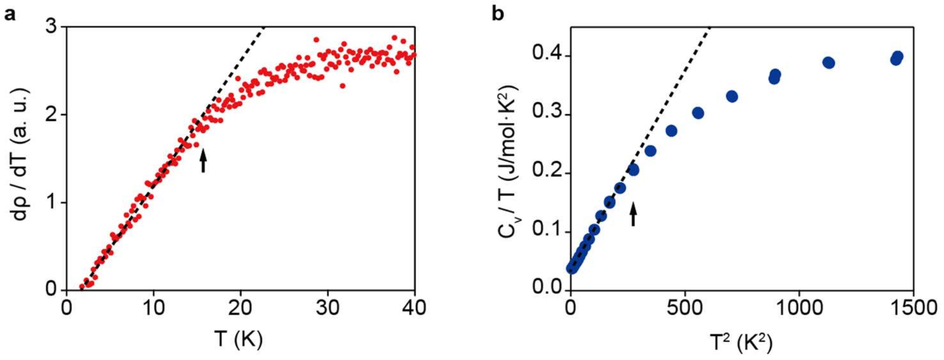

Supplementary Fig. 1. Coherent-incoherent crossover seen in transport measurements. a. Temperature derivative of resistivity. The black dashed line is a guide to the eye. **b**. Temperature-dependent C_V/T.

Precise analyses of transport results in Supplementary Fig. 1 show the coherence temperature to be around 15 K at which the resistivity and heat capacity deviate from Fermi liquid behavior.

Supplementary Note 2: Resistivity of FeTe at low temperatures

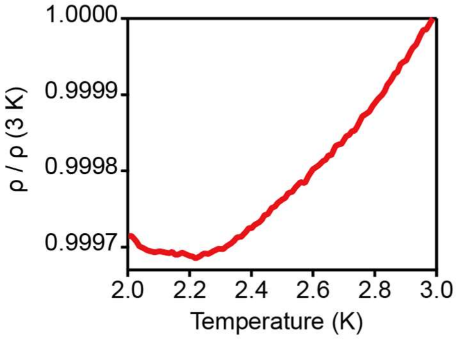

Supplementary Fig. 2. Resistivity of FeTe at low temperatures.

Precise resistivity measurements at low temperatures exhibit a resistivity minimum at around 2.2 K as shown in Supplementary Fig. 2, which may be attributed to the Kondo effect induced by excess Fe. However, we would like to note that this is different from the Kondo lattice behavior at a much higher temperature scale discussed in the manuscript.

Supplementary Note 3: Temperature-dependent ARPES measurements at the X point pocket.

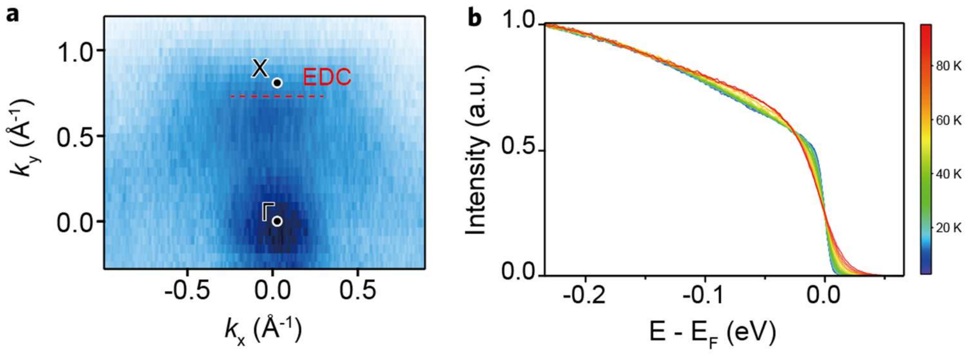

Supplementary Fig. 3. Temperature evolution of X-point pocket. a. Fermi surface map measured at 6 K. **b**. Extracted temperature-dependent energy distribution curves (EDCs) at the red dashed line in **a**.

To figure out whether the Γ pocket seen in Fig. 2 exclusively determines the transport properties of FeTe, we additionally conducted ARPES measurements near the X point region. We note that the M point pocket of FeTe shows negligible spectral weight near the Fermi level while that near the X point shows a rather strong spectral weight[1,2]. Supplementary Fig. 3a shows the Fermi surface taken with 21 eV light. As can be seen, the Femi surface map shows relatively strong spectral weight near the X point. Temperature dependence of energy distribution curves (EDCs) at the X point is shown in Supplementary Fig. 3b. Contrary to the Γ point data, which show a strong temperature dependence and a quasiparticle peak (QP) at low temperature, there is no significant temperature dependence and no signature of quasiparticle peak at the X point. The spectra near the Fermi level follow a usual Fermi-Dirac distribution. It clearly shows that the temperature dependent behavior at the X point is not consistent with the transport properties across T_N. A drastic change in transport properties across the magnetic transition should be accompanied by a change in the Femi surface and corresponding QP, which is not the case for the X point pocket. Taking these points into account, we would like to point out that Γ point pocket dominates the Kondo behavior observed by transport and STM measurements.

Supplementary Note 4: Fitting functions and detailed parameters of Fano function

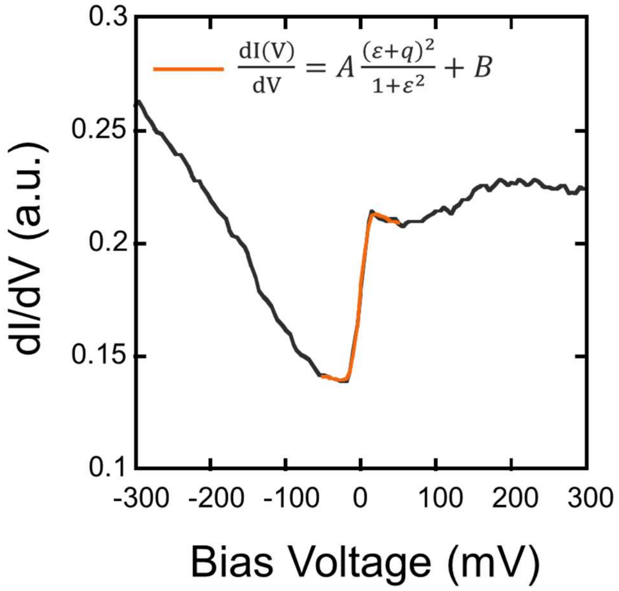

Supplementary Fig. 4. Fano fitting function and fitting results

We have fitted the dI/dV spectrum measured at 4.3 K to the Fano function of $\frac{dI(V)}{dV} = A\frac{(\varepsilon+q)^2}{1+\varepsilon^2} + B$ as illustrated in Supplementary Fig. 4[3-6]. Here, $\varepsilon = (\omega - \varepsilon_0)/\Gamma$, $\omega = eV$ ε_0 is the resonance energy, q is the Fano interference parameter, and Γ (= $k_B T_k$) is half-width at half-maximum of Kondo peak. We found the fitting parameters q = -0.92, ε_0 = 0.1, A = -0.033, Γ = 24.1 meV and B = 0.2 reproduce the resonant spectrum very well. Considering the relationship between Γ value and Kondo temperature ($k_B T_K = \Gamma$), the estimated Kondo temperature is around 281 K. This value roughly coincides with the onset temperature of Kondo scattering from resistivity, which is around room temperature.

Supplementary Note 5: Topographic image and real-space dependence of STS results

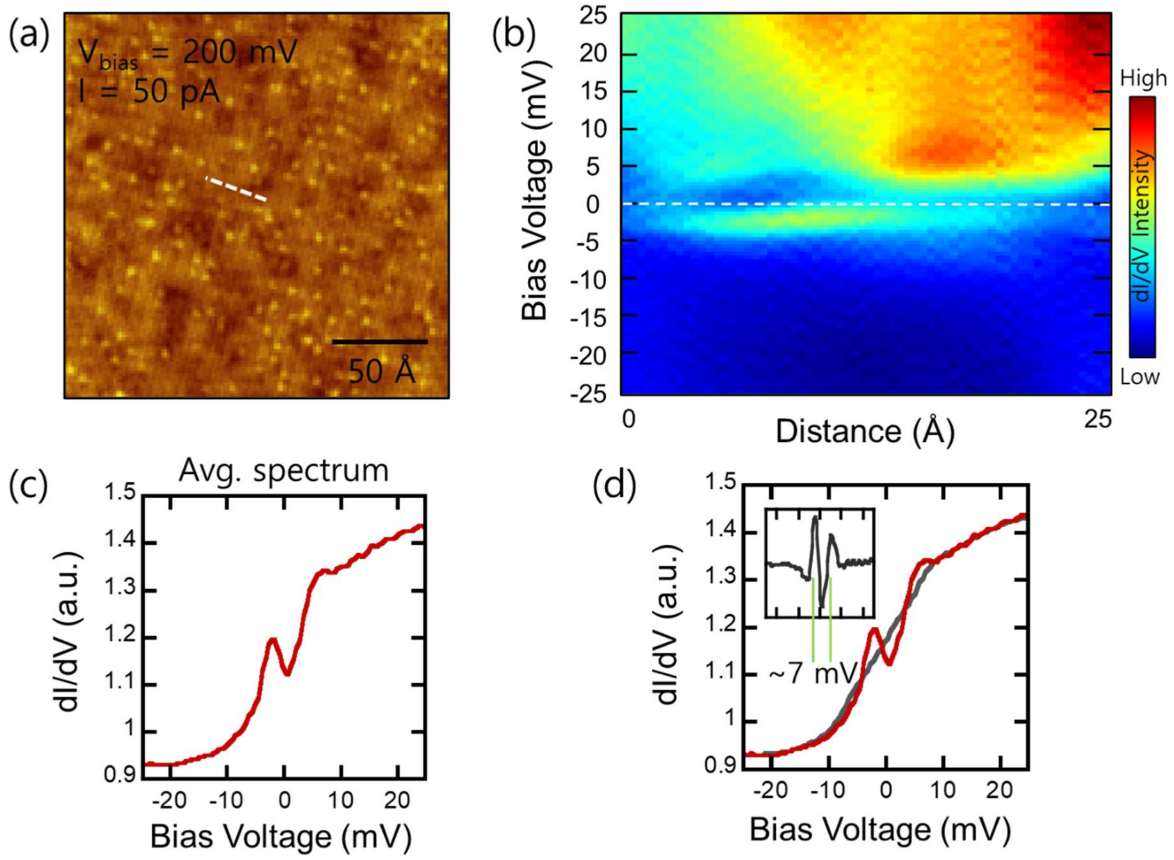

Supplementary Fig. 5. a. Topographic image of $Fe_{1.1}Te$ taken with V_{bias} = 200 mV and I = 50 pA. **b.** dI/dV spectra measured along the dotted line in **a**. 64 spectra were taken. V_{bias} = -40 mV, I = 50 pA and V_{mod} = 500 mV$_{pp}$. **c.** A spectrum obtained by averaging the 64 spectra. **d.** The gray curve represents that the smoothly-varying background which is obtained by Gaussian-smoothing the measured dI/dV spectrum. The inset shows the spectrum after subtracting the background spectrum. The gap size is approximately 7 mV.

Supplementary Note 6: Temperature-dependent STS results

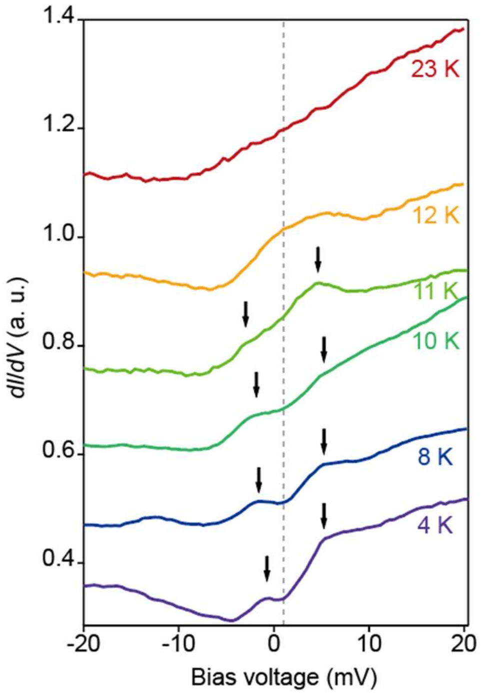

Supplementary Fig. 6. Temperature-dependent STS results. Spectra are offset arbitrarily for better visualization. The gray dashed line denotes the gap position estimated from the 4 K data and black arrows indicate the peak position originated from the gap feature.

Supplementary Note 7: Extended magnetic phase diagram calculated from the model Hamiltonian

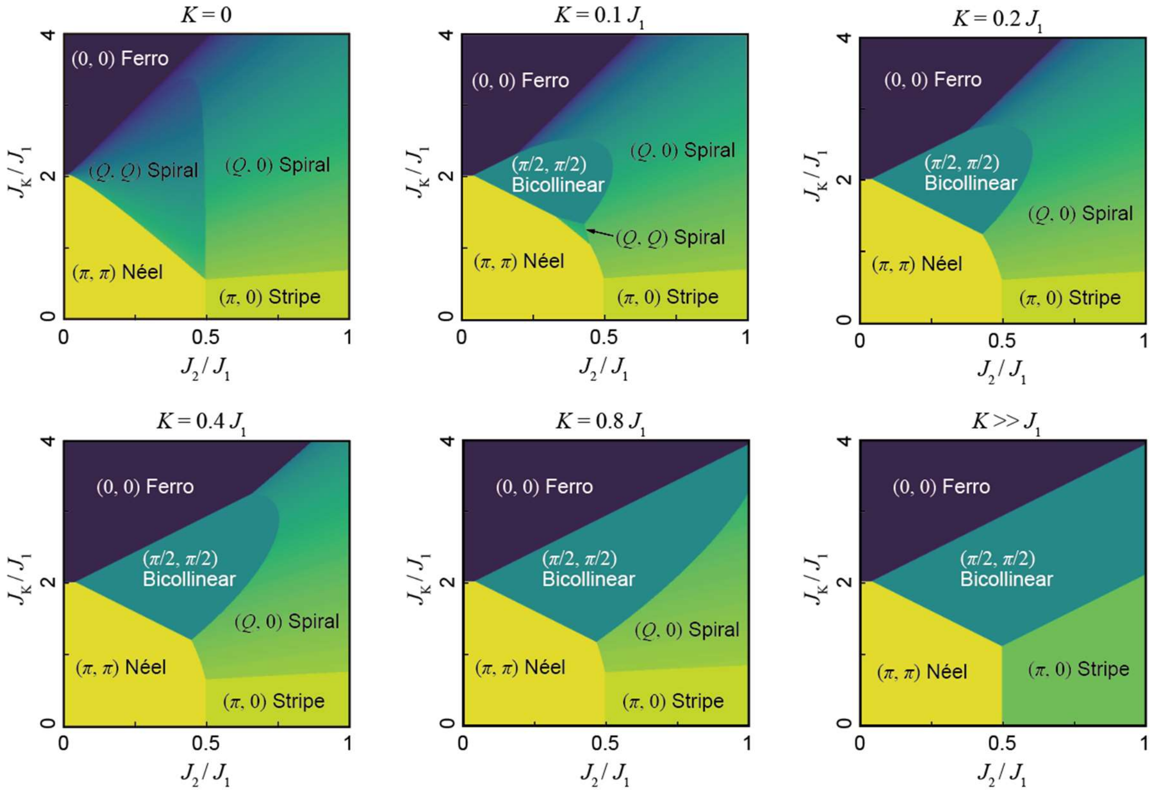

Supplementary Fig. 7. Extended magnetic phase diagram of FeTe from Heisenberg model.

Supplementary Note 8: Density functional theory (DFT) calculations on FeTe

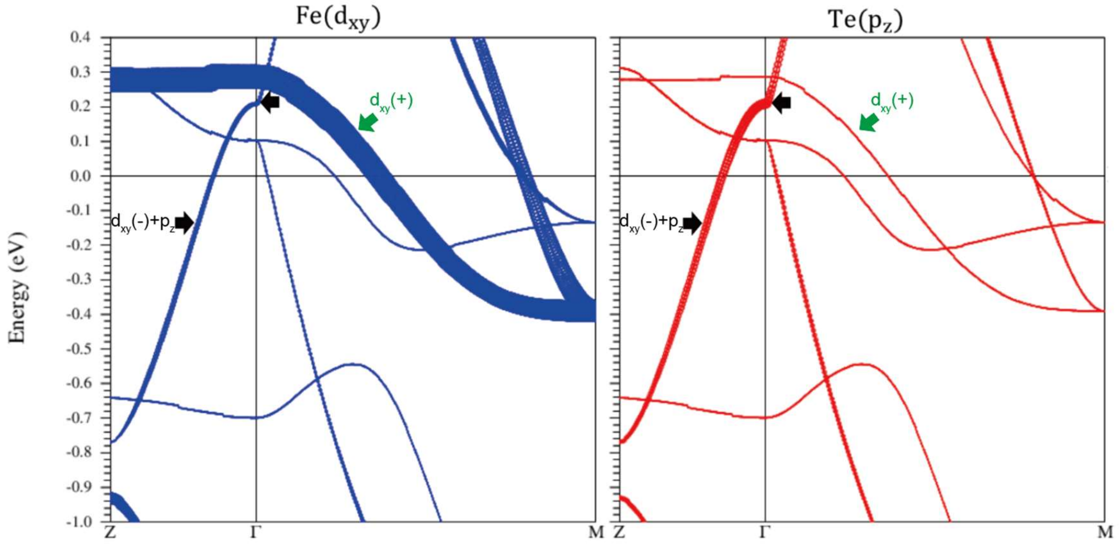

Supplementary Fig. 8. Density functional calculation results on FeTe. The obtained electronic structure is projected on Fe d_{xy} (left) and Te p_z (right). The black arrows indicate the band which corresponds to the dispersive band measured by ARPES. $d_{xy}(+)$ and $d_{xy}(-)$ denote even and odd parity dxy band for the inversion symmetry, respectively. The thickness of the bands represents the weight of projected orbital characters.

To investigate the orbital character of the band which hybridized with Te p_z orbitals, we additionally conducted DFT-LDA calculations on FeTe based on experimentally obtained crystal structures[9]. Shown in Supplementary Fig. 8 are DFT calculation results projected on Fe d_{xy} and Te pz. The thickness of bands indicates a projected weight of the corresponding orbital. The band indicated by black arrows corresponds to what we have measured by ARPES. The band has a strong Te p_z character as well as Fe d_{xy} character, implying that the two orbitals are strongly hybridized. From these results, we can confirm that the experimentally measured band has Fe d_{xy} orbital character as well as Te p_z orbital character.

Supplementary Note 9: Dynamic mean field theory (DMFT) calculations on FeTe

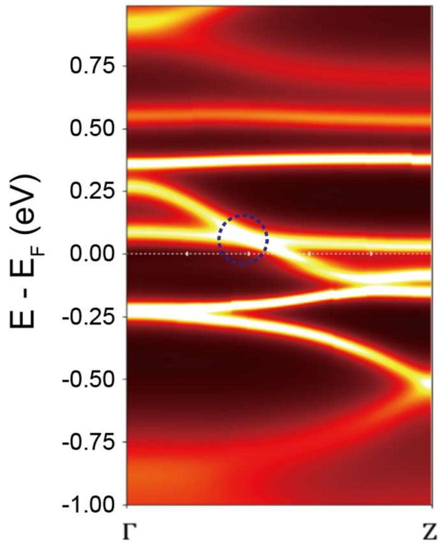

Supplementary Fig. 9. Dynamic mean field theory (DMFT) calculations on FeTe.

Shown in Supplementary Fig. 9 is the DMFT calculation result along the Γ-Z line. The magnetic ground state in the DMFT calculation successfully converged to the bicollinear antiferromagnetic state. The strongly dispersive band (p_z) and strongly localized band cross each other near the Fermi level as indicated by the blue dashed circle. This is an indication of the Kondo hybridization, where the localized and itinerant bands cross each other near the Fermi level, even though we could not observe the hybridization gap due to the finite broadening effect of the DMFT calculations.

Supplementary Note 10: Geometry-, and polarization-dependent Fermi surfaces of FeTe

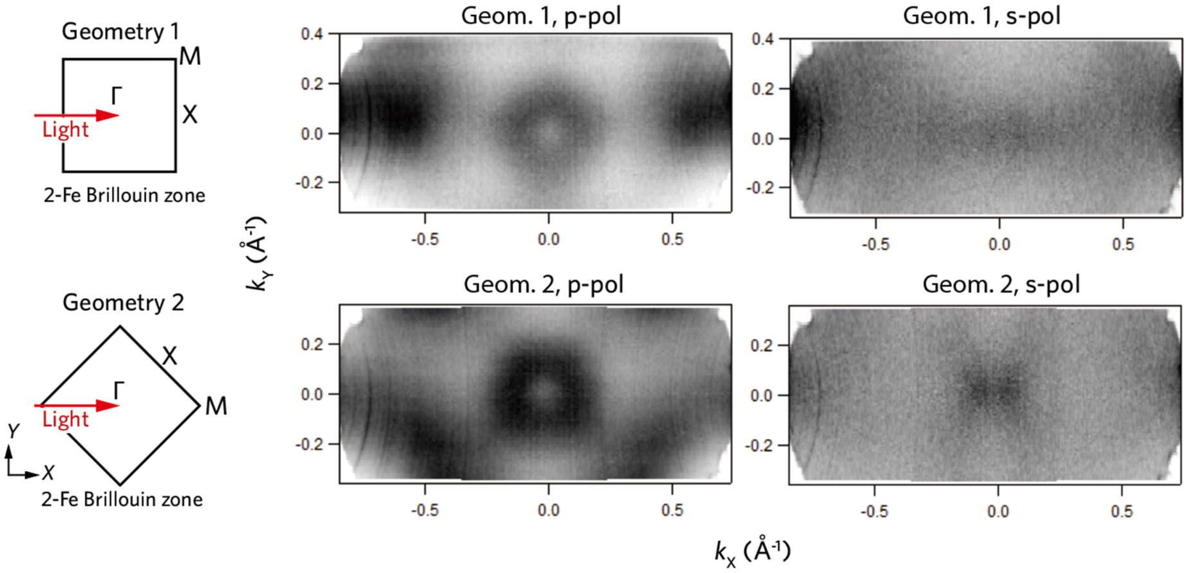

Supplementary Fig. 10. Geometry- and polarization-dependent FeTe Fermi surface. ΓM(ΓX) denotes the geometry in which the ΓM(ΓX) line of the sample is aligned along the incident photon. The ARPES data are taken with 29 eV light. The X and Y directions are defined in the laboratory frame.

Geometry 1	*p*-pol	*s*-pol	Geometry 2	*p*-pol	*s*-pol
d_{xz}	O	O	d_{xz}	O	X
d_{yz}	O	O	d_{yz}	X	O
d_{xy}	O	X	d_{xy}	X	O
p_z	O	X	p_z	O	X

Supplementary Table 1. Sample geometry and polarization dependent photoemission selection rules. Geometry 1(2) denotes the geometry in which the ΓX(ΓM) line of the sample is aligned along the incident photon. Here, the orbitals are defined in the sample frame.

Summarized in Supplementary Table 1 are the photoemission selection rules of t_{2g} and p_z orbitals near the Γ point as a function of the sample geometry and light polarization[8,10]. Here, Geometry 1(2) represents the geometry in which the ΓX (ΓM) line of the sample is aligned along the incident photon (see the schematic of the sample geometry on the left side of Supplementary Fig. 10). Since the orbital character of the electron band can be

determined based on the selection rules presented in Supplementary Table 1, we performed polarization-, geometry-dependent ARPES to experimentally determine the orbital character.

Supplementary Fig. 10 shows that a circular electron pocket is only visible with p-polarization, regardless of sample geometry. Based on the selection rules given in Table R1, such experimental polarization dependence is possible only for the p_z orbital.

References

[1] J. Jiang, C. He, Y. Zhang, M. Xu, Q. Q. Ge, Z. R. Ye, F. Chen, B. P. Xie, and D. L. Feng, Physical Review B 88 (2013).

[2] Z. K. Liu et al., Physical Review B 92 (2015).

[3] T. Uchihashi, J. Zhang, J. Kröger, and R. Berndt, Phys. Rev. B **78**, 033402 (2008).

[4] P. Wahl, A. P. Seitsonen, L. Diekhöner, M. A. Schneider and K. Kern, New J. Phys. **11**, 113015 (2009).

[5] J. Bork et al., Nat. Phys. **7**, 901-906 (2011).

[6] S. Meierott, N. Néel, and J. Kröger, Phys. Rev. B **91**, 201111(R) (2015).

[7] Wang, Z. et al. Physical Review B 92, Phys. Rev. B **92**, 115119 (2015).

[8] Zhang, P. et al. Science **360**, 182-186, science.aan4596 (2018).

[9] Mizuguchi, Y., et al., Physica C **469**, 1027-1029 (2009).

[10] Sobota, J. A., He, Y., Shen, Z.-X., Reviews of Moden Physics **93**, 025006 (2021).
